# Synthesis, crystal structure and Hirshfeld surface analysis of bis­(acetyl­acetonato-κ^2^*O*,*O*′)(2-amino-1-methyl-1*H*-benzimidazole-κ*N*^3^)copper(II)

**DOI:** 10.1107/S2056989024011538

**Published:** 2025-01-01

**Authors:** Kyzlarkhan Siddikova, Daminbek Ziyatov, Akmaljon Tojiboev, Jamshid Ashurov, Zukhra Kadirova, Shahlo Daminova

**Affiliations:** a Karshi Engineering Economics Institute, Mustakillik Avenue 225, Karshi 180100, Uzbekistan; b National University of Uzbekistan named after Mirzo Ulugbek University Street 4 Tashkent 100174 Uzbekistan; c University of Geological Sciences, Olimlar Street 64, Tashkent 100125, Uzbekistan; d Namangan State University, Boburshox Street 161, Namangan 160107, Uzbekistan; e Institute of Bioorganic Chemistry, Academy of Sciences of Uzbekistan, Mirzo Ulugbek Street 83, Tashkent 100125, Uzbekistan; f Uzbek-Japan Innovation Center of Youth, University Street 2B, Tashkent 100095, Uzbekistan; University of Neuchâtel, Switzerland

**Keywords:** benzimidazole, acetyl­acetone, copper complex, crystal structure, Hirshfeld surface analysis

## Abstract

The title compound, [Cu(C_5_H_7_O_2_)_2_(C_8_H_9_N_3_)], crystallizes in the ortho­rhom­bic space group *Pnma*. In the crystal structure, the Cu^II^ ion is coordinated by two acetyl­acetonate ligands and one 2-amino-1-methyl-1*H*-benzimidazole ligand. The crystal structure features intra­molecular N—H⋯O and inter­molecular N—H⋯O hydrogen bonds, which contribute to the overall cohesion of the crystal. Hirshfeld surface analysis and two-dimensional fingerprint plots were utilized to qu­antify the inter­molecular inter­actions, revealing the relative contributions of H⋯H (61.1%), H⋯C/C⋯H (21.3%), and O⋯H/H⋯O (11.3%) contacts to the crystal packing.

## Abstract 2

A compound of composition [Cu(C_5_H_7_O_2_)_2_(C_8_H_9_N_3_)] was obtained. In the crystal, the Cu^II^ ion exhibits a square-pyramidal geometry with the ligands (two acetyl­acetone and one 2-amino-1-methyl­benzimidazole). Hirshfeld surface analysis was used to investigate the inter­molecular inter­actions.

## 1. Chemical context

Transition-metal complexes containing Schiff base ligands have garnered significant attention in recent years due to their promising catalytic activity in various reactions (Sheikhshoaie *et al.*, 2009 ▸; Hatefi *et al.*, 2010 ▸; Rezaeifard *et al.*, 2010 ▸). Benzimidazole derivatives have also attracted considerable inter­est due to their diverse biological and therapeutic activities, including anti­microbial properties against bacteria such as methicillin-resistant *staphylococcus aureus* (Gatadi *et al.*, 2019 ▸), *escherichia coli* (Mishra *et al.*, 2019 ▸), and *bacillus subtilis* (Song & Ma, 2016 ▸). The discovery of new benzimidazole compounds with novel anti­bacterial mechanisms is of paramount importance in addressing the growing threat of anti­biotic resistance (Khalafi-Nezhad *et al.*, 2005 ▸). Recent research has focused on the synthesis and characterization of benzimidazole-based complexes with *d*-block metals (Jabborova *et al.*, 2024 ▸) and *f*-block metals (Ruzieva *et al.*, 2022 ▸), further expanding the potential applications of these compounds.

The coordination chemistry of rare-earth metals with β-diketonate ligands has been extensively studied due to their versatility and ease of use (Binnemans, 2005 ▸). β-Dicarbonyl compounds, known for their keto–enol tautomerism, are among the most widely investigated tautomeric systems (Tighadouini *et al.*, 2022 ▸; Harris, 2001 ▸). Metal acetyl­acetonates have found applications in diverse fields, including redox flow batteries (Suttil *et al.*, 2015 ▸) and as corrosion inhibitors for mild steel (Mahdavian & Attar, 2009 ▸). Notably, Cu^II^, Ni^II^, Co^II^, and Zn^II^ complexes with acetyl­acetone ligands have demonstrated enhanced anti­microbial activity compared to the free ligand (Raman *et al.*, 2003 ▸). In light of the potential biological significance of the title compound, [Cu(C_5_H_7_O_2_)_2_(C_8_H_9_N_3_)], a detailed investigation of its crystal structure is presented.
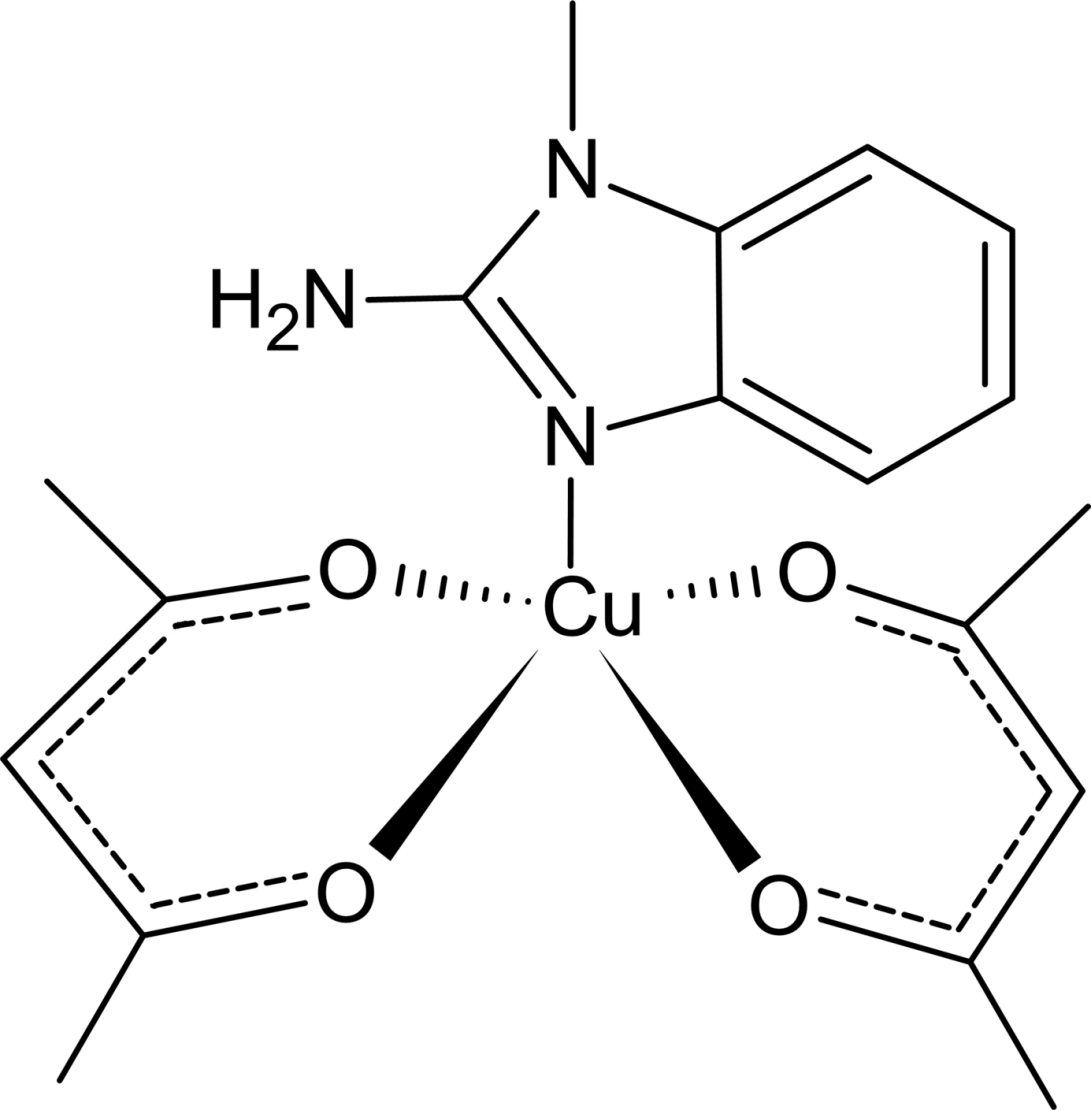


## 2. Structural commentary

**Figure 1:**
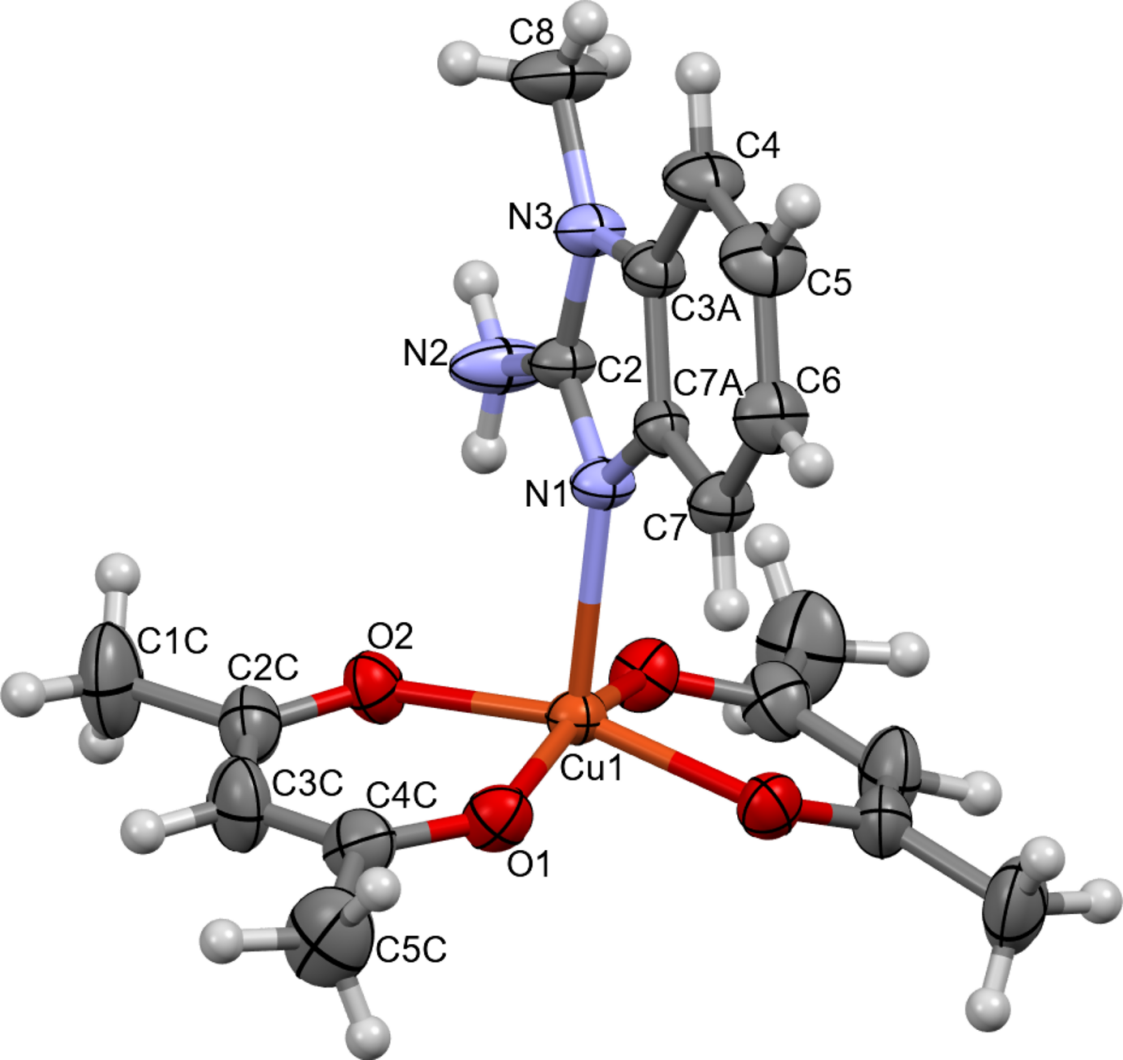
Mol­ecular structure of the title compound. Displacement ellipsoids are drawn at the 30% probability level. The hydrogen atoms and symmetry-generated atoms are not labeled.

**Figure 2:**
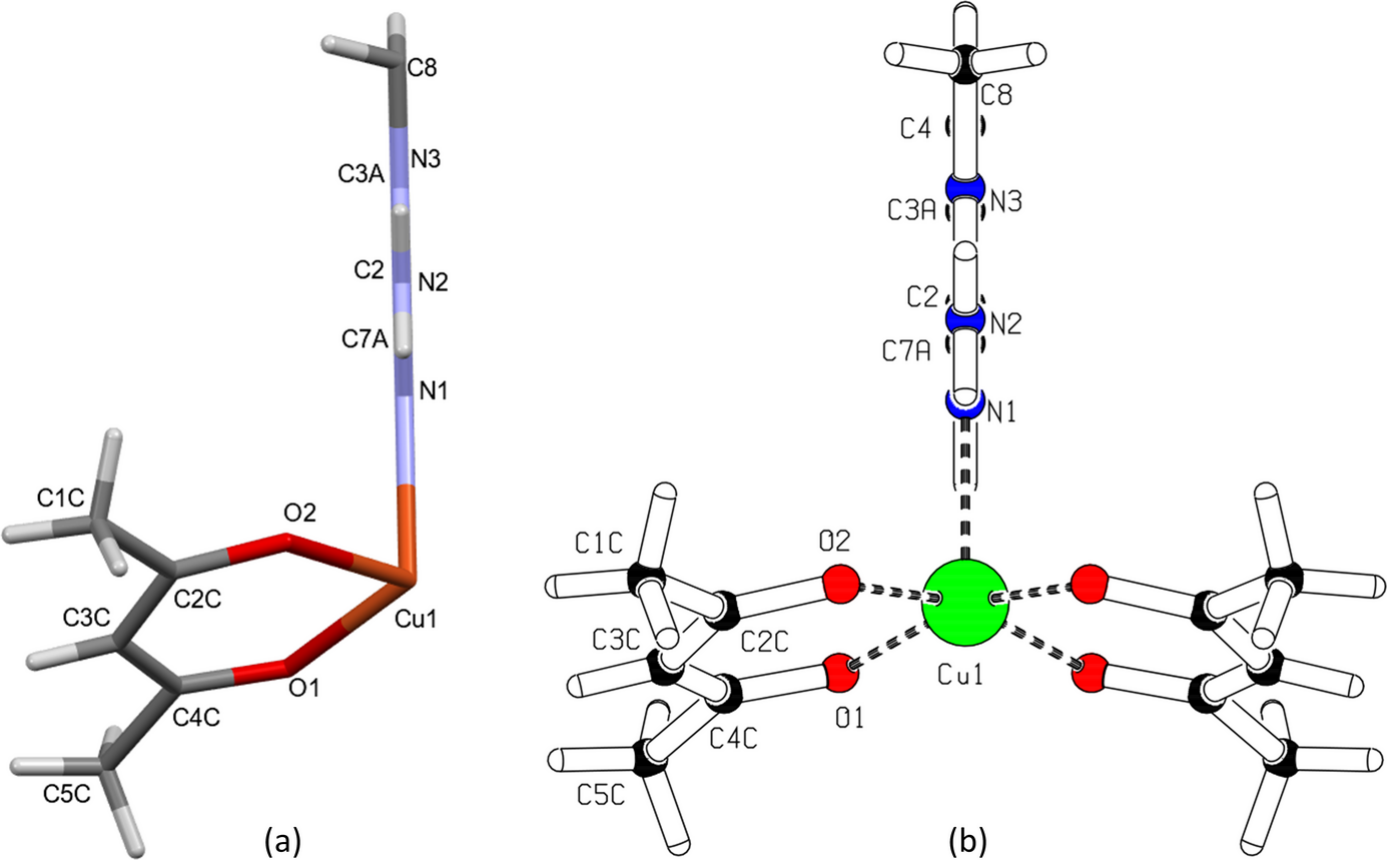
(*a*) The asymmetric unit in the crystal of **I**. (*b*) Emphasis on the benzimidazole planarity.

The title compound, bis­(acetyl­acetonato-κ^2^*O*,*O*′)(2-amino-1-methyl-1*H*-benzimidazole-κ*N*^3^)copper(II) (**I**), crystallizes in the ortho­rhom­bic space group *Pnma* (Fig. 1 ▸). The asymmetric unit consists of one mol­ecule of 2-amino-1-methyl-1*H*-benzimidazole (**MAB**) and one acetyl­acetonate (**acac**) ligand, both coordinated to the central copper(II) ion. The Cu^II^ ion adopts a square-pyramidal coordination geometry (coordination number 5), with the equatorial plane defined by the oxygen atoms of two bidentate β-diketonate mol­ecules [Cu1—O1 = 1.9378 (16) Å; Cu1—O2 = 1.9546 (16) Å; Table 1 ▸]. The observed elongation of the Cu—O bonds is attributed to the Jahn–Teller effect, a common phenomenon in copper-based complexes (Halcrow, 2013 ▸). The benzimidazole moiety is essentially planar and lies in the plane of symmetry (Fig. 2 ▸). The N1 atom of the benzimidazole ligand coordinates axially to the Cu^II^ ion with a bond distance of 2.196 (2) Å. The observed Cu—N1, Cu—O1, and Cu—O2 bond lengths are consistent with those reported for related Cu^II^ complexes (Geiger *et al.*, 2017 ▸; Wong *et al.*, 2009 ▸). The root-mean-square deviation of the equatorial plane (defined by O1, O2, Cu1, and their symmetry-related counterparts O1^i^ and O2^i^) is 0.118 Å, with out-of-plane distances of 0.0596 (16) Å for O1 and 0.0582 (16) Å for O2. The largest deviation from the plane is observed for the Cu^II^ ion [0.2357 (4) Å], which is attributed to the presence of only one axial ligand (Fig. 1 ▸). The mol­ecular structure of **I** exhibits intra­molecular N—H⋯O hydrogen bonds (Table 2 ▸), which contribute to the stability of the individual mol­ecules. These hydrogen bonds form a characteristic 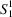
(6) graph-set motif (Etter *et al.*, 1990 ▸).

## 3. Supra­molecular features

**Figure 3:**
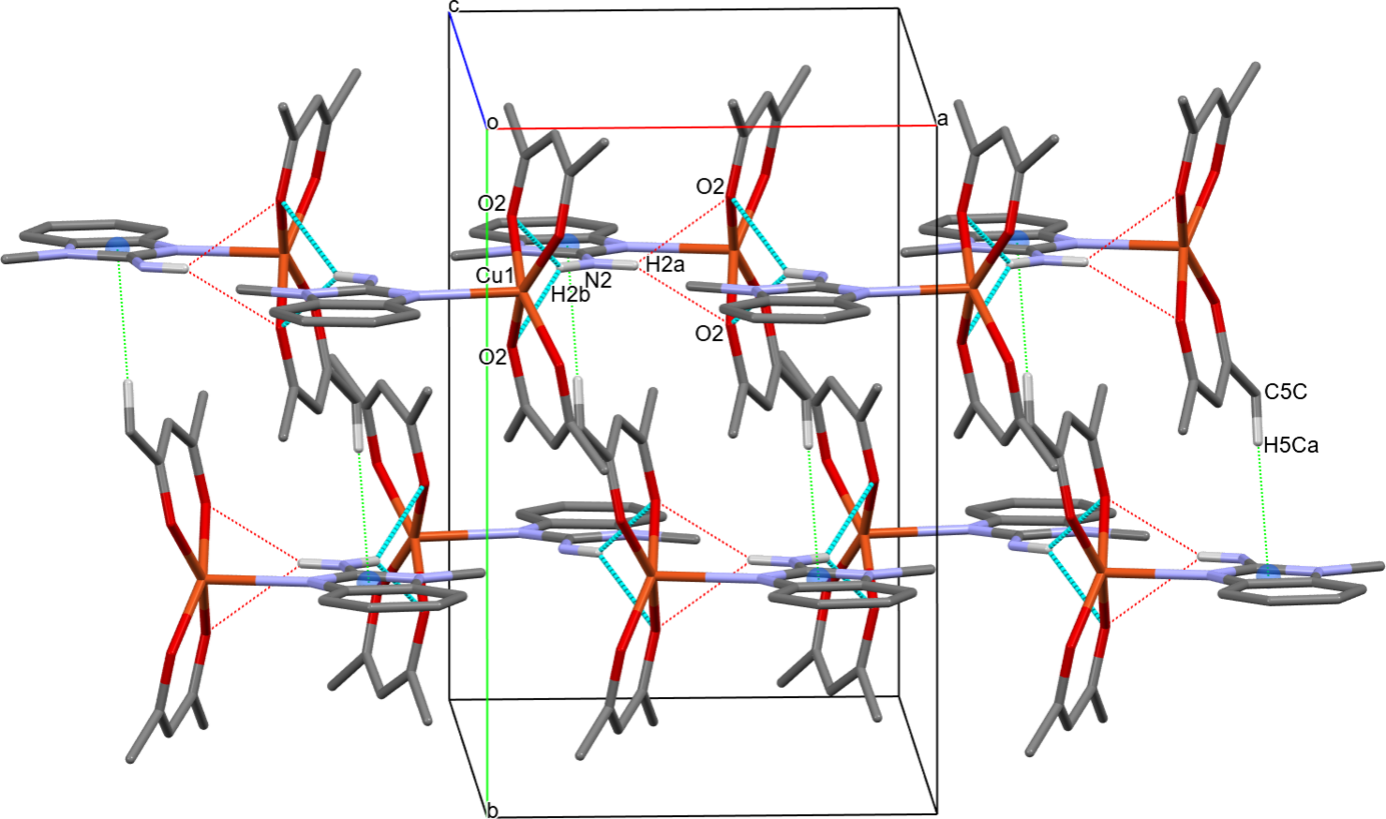
Crystal packing of **I** along the *c* axis. Intra­mol­ecular N—H⋯O hydrogen bonds are shown as red and inter­mol­ecular N—H⋯O hydrogen bonds as light-blue dashed lines. Dashed green lines denote C—H⋯*Cg*1 contacts. For clarity, H atoms not involved in these inter­actions have been omitted.

Inter­molecular N—H⋯O hydrogen bonds play a crucial role in establishing the overall crystal packing. These inter­molecular inter­actions link the mol­ecules into a zigzag chain running along the crystallographic a-axis direction, as depicted in Fig. 3 ▸. The graph-set descriptors for these chains are 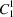
(6) and 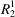
(4), further illustrating the connectivity of the hydrogen-bonded network.

The structure also features π-ring inter­actions between adjacent chains, which contribute to the overall cohesion of the crystal. These inter­actions involve C—H⋯π contacts, where the C5—H5*C* bond of one mol­ecule inter­acts with the centroid (*Cg*1) of the N1/C2/N3/C3*A*/C7*A* ring of a neighbouring mol­ecule. The distance between the hydrogen atom (H5*C*) and the ring centroid (*Cg*1) is 2.743 (16) Å, indicating a significant inter­action.

The combination of intra­molecular and inter­molecular hydrogen bonds, along with π-ring inter­actions, results in a robust three-dimensional supra­molecular network in the crystal structure of **I**. These inter­actions not only contribute to the overall cohesion of the crystal but may also influence the physical and chemical properties of the compound.

## 4. Hirshfeld surface analysis

**Figure 4:**
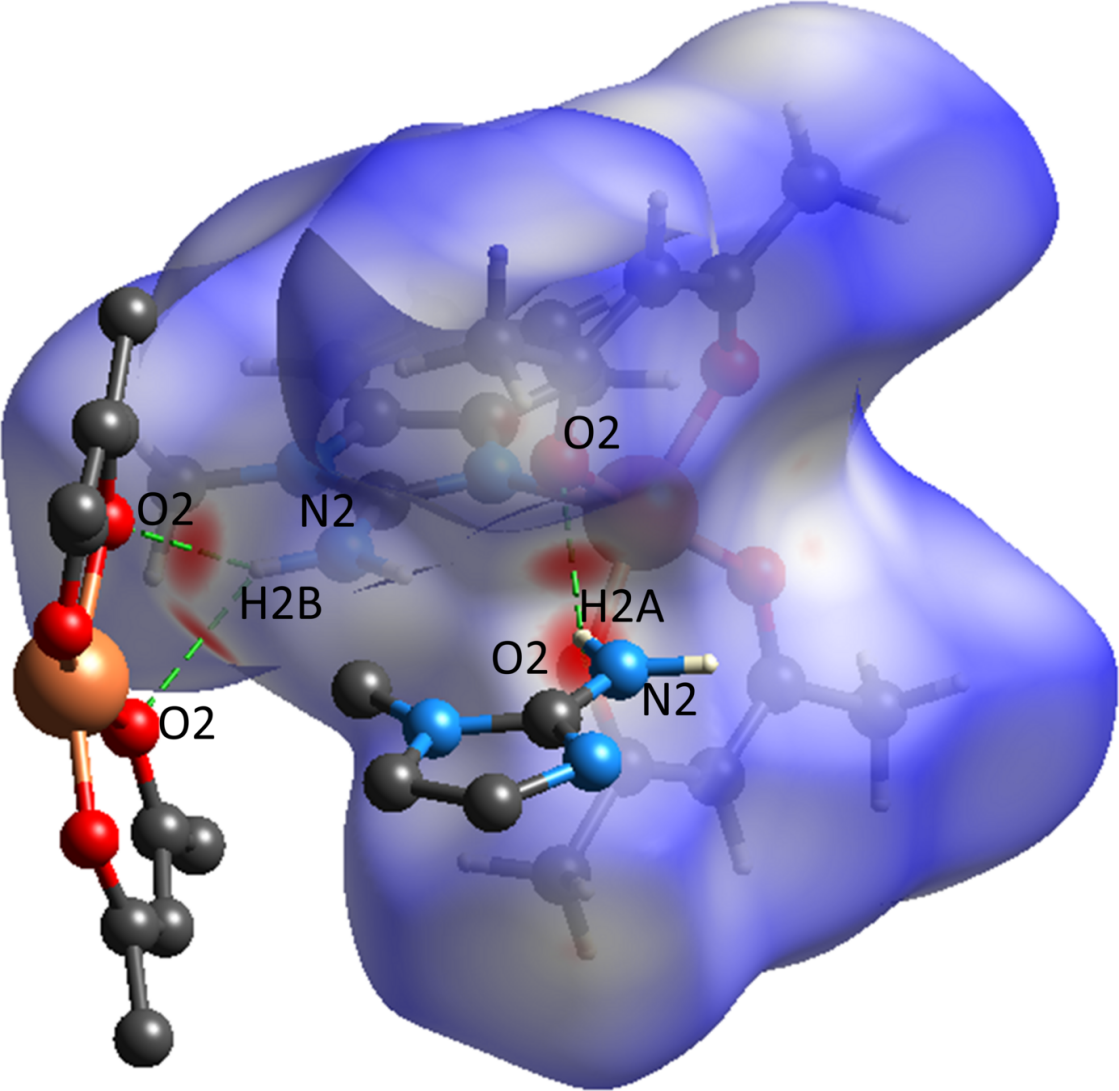
View of the three-dimensional Hirshfeld surface of **I** plotted over *d*_norm_.

Hirshfeld surface analysis was conducted using *CrystalExplorer* 21.5 (Spackman *et al.*, 2021 ▸) to gain further insights into the inter­molecular inter­actions in the crystal structure of **I**. The *d*_norm_ surface, shown in Fig. 4 ▸, is mapped over −0.2120 to −1.5316 arbitrary units (a.u.), with red, white, and blue regions representing contacts shorter, equal to, or longer than the sum of van der Waals radii, respectively(Venkatesan *et al.*, 2016 ▸).

**Figure 5:**
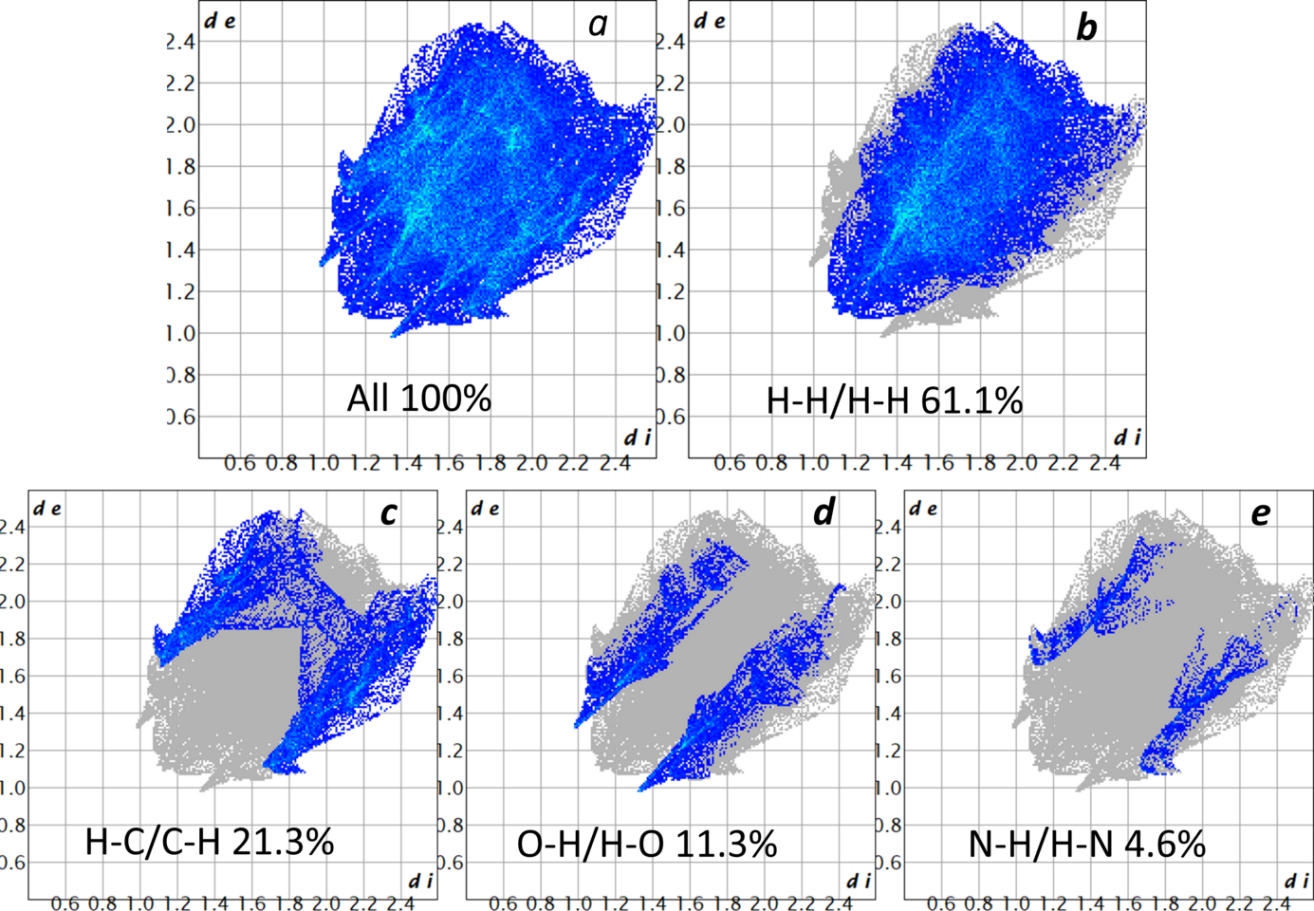
The full two-dimensional fingerprint plots for **I**, showing (*a*) all inter­actions and (*b*)–(*d*) delineated into separate inter­actions.

The overall two-dimensional fingerprint plot (Fig. 5 ▸*a*) and its decomposed components illustrate the relative contributions of different inter­molecular contacts to the Hirshfeld surface. As expected, H⋯H contacts (Fig. 5 ▸*b*) constitute the most significant contribution, accounting for 61.1% of the total Hirshfeld surface area. H⋯C/C⋯H contacts (Fig. 5 ▸*c*) comprise 21.3%, followed by O⋯H/H⋯O contacts (Fig. 5 ▸*d*) at 11.3%. The remaining contributions, including N⋯H/H⋯N (4.6%), Cu⋯C (1.0%), and C⋯C (0.7%), are relatively minor. These results highlight the dominance of van der Waals inter­actions in the crystal packing of **I**.

## 5. Database survey

A search of the Cambridge Structural Database (CSD, Version 5.45, November 2023; Groom *et al.*, 2016 ▸) revealed two related structures: bis­(acetyl­acetonato-κ^2^*O*,*O*′)(2-amino-1-methyl-1*H*-benzimidazole-κ*N*)oxidovanadium(IV) (Kadi­rova *et al.*, 2009 ▸; CSD refcode BOVMAB) and aqua­(benzimidazole-*N*)bis­(2,4-penta­nedionato-*O*,*O*′)cobalt(II) (Lin & Feng, 2003 ▸; CSD refcode ESUZUN). In both cases, the benzimidazole ligand coordinates to the central metal ion through the *sp*^2^ nitro­gen atom (N3), as observed in **I**. However, the coordination geometries and overall structural features differ due to the presence of different metal centers and additional ligands in these related complexes.

## 6. Synthesis and crystallization

All reagents and solvents were of analytical grade and used as received. Elemental analysis was performed using a FlashSmart™ Elemental Analyzer. The Fourier-transform infrared (FT–IR) spectrum was recorded on a Spectrum Two N FT–IR Spectrometer at room temperature.

Solutions of 0.1 mmol (0.0261 g) of CuCl_2_·6H_2_O in ethanol (solution *A*), 0.2 mmol (0.0294 g) of 2-amino-1-methyl-1*H*-benzimidazole (MAB) in ethanol (solution *B*), and 0.2 mmol (0.0205 mL, ρ = 0.975 g mL^−1^) of acetyl­acetone (solution *C*) were prepared. Solution *B* was added dropwise to solution *A* with stirring at room temperature for 30 minutes; no immediate changes being observed. Subsequently, solution *C* was added dropwise to the mixture, followed by stirring for 12 h. The resulting solution was then allowed to stand undisturbed at room temperature. Blue–green crystals formed over several days, which were then filtered, washed with ethanol, and recrystallized from dimethyl sulfoxide to yield light-green crystals suitable for X-ray diffraction analysis.

**Elemental analysis:** Calculated for C_18_H_23_CuN_3_O_4_: C, 52.88; H, 5.67; N, 10.28%. Found: C, 53.06; H, 5.33; N, 10.59%.

**FT–IR (cm^−1^):** 3448*s*, 3341*s*, 3054*s*, 2935*m*, 1654*s*, 1612*s*, 1584*s*, 1551*s*, 1522*s*, 1499*s*, 1399*s*, 1319*s*, 1289*s*, 1200*m*, 1108*s*, 1089*m*, 1018*s*, 934*s*, 894*m*, 787*s*, 746*s*, 674*m*, 657*s*, 587*s*, 566*m*, 429*m*.

## 7. Refinement

Crystal data, data collection, and structure refinement details are summarized in Table 3 ▸. Hydrogen atoms bonded to carbon were positioned geometrically (C—H = 0.93 Å for aromatic, 0.96 Å for methyl, and 0.97 Å for methyl­ene) and refined using a riding model, with *U*_iso_(H) = 1.5*U*_eq_(C) for methyl hydrogens and 1.2*U*_eq_(C) for all others. The amine group (–NH_2_) hydrogen atoms were located in a difference-Fourier map and refined with an N—H distance restraint of 0.86 (2) Å and *U*_iso_(H) = 1.5*U*_eq_(N).

## Supplementary Material

Crystal structure: contains datablock(s) I. DOI: 10.1107/S2056989024011538/tx2089sup1.cif

Structure factors: contains datablock(s) I. DOI: 10.1107/S2056989024011538/tx2089Isup2.hkl

CCDC reference: 2405560

Additional supporting information:  crystallographic information; 3D view; checkCIF report

## Figures and Tables

**Table 1:** Selected bond lengths (Å)

Cu1—O1^i^	1.9378 (16)	Cu1—O2	1.9546 (16)
Cu1—O1	1.9378 (16)	Cu1—O2^i^	1.9546 (16)
Cu1—N1	2.196 (2)		

Symmetry code: (i) 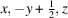.

**Table 2:** Hydrogen-bond geometry (Å, °) *Cg*1 is the centroid of the N1/C2/N3/C3*A*/C7*A* ring.

*D*—H⋯*A*	*D*—H	H⋯*A*	*D*⋯*A*	*D*—H⋯*A*
N2—H2*A*⋯O2	0.86	2.44	3.077 (3)	131
N2—H2*B*⋯O2^ii^	0.86	2.44	3.167 (3)	142
C5*B*—H5*BA*⋯*Cg*1^iii^	0.96	2.74	3.682 (4)	166

Symmetry codes: (ii) 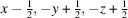; (iii) 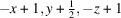.

**Table 3:** Experimental details

Crystal data	
Chemical formula	[Cu(C_5_H_7_O_2_)_2_(C_8_H_9_N_3_)]
*M*_r_	408.93
Crystal system, space group	Orthorhombic, *Pnma*
Temperature (K)	298
*a*, *b*, *c* (Å)	9.0322 (2), 13.9740 (3), 16.0004 (4)
*V* (Å^3^)	2019.51 (8)
*Z*	4
Radiation type	Cu *K*α
μ (mm^−1^)	1.70
Crystal size (mm)	0.3 × 0.24 × 0.18

Data collection	
Diffractometer	XtaLAB Synergy, Single source at home/near, HyPix3000
Absorption correction	Multi-scan (*CrysAlis PRO*; Rigaku OD, 2020 ▸)
*T*_min_, *T*_max_	0.517, 1.000
No. of measured, independent and observed [*I* ≥ 2σ(*I*)] reflections	19729, 2050, 1693
*R*_int_	0.066
(sin θ/λ)_max_ (Å^−1^)	0.615

Refinement	
*R*[*F*^2^ > 2σ(*F*^2^)], *wR*(*F*^2^), *S*	0.036, 0.109, 1.04
No. of reflections	2050
No. of parameters	146
H-atom treatment	H atoms treated by a mixture of independent and constrained refinement
Δρ_max_, Δρ_min_ (e Å^−3^)	0.27, −0.34

Computer programs: *CrysAlis PRO* (Rigaku OD, 2020 ▸), *SHELXT2014/5* (Sheldrick, 2015 ▸), *OLEX2.refine* (Bourhis *et al.*, 2015 ▸) and *OLEX2* (Dolomanov *et al.*, 2009 ▸).

## supplementary crystallographic information

### Bis(acetylacetonato-κ2O,O')(2-amino-1-methyl-1H-\ benzimidazole-κN3)copper(II) Crystal data

**Table:**

[Cu(C_5_H_7_O_2_)_2_(C_8_H_9_N_3_)]	*D*_x_ = 1.345 Mg m^−^^3^
*M**_r_* = 408.93	Cu *K*α radiation, λ = 1.54184 Å
Orthorhombic, *P**n**m**a*	Cell parameters from 5665 reflections
*a* = 9.0322 (2) Å	θ = 2.8–69.5°
*b* = 13.9740 (3) Å	µ = 1.70 mm^−^^1^
*c* = 16.0004 (4) Å	*T* = 298 K
*V* = 2019.51 (8) Å^3^	Block, clear light green
*Z* = 4	0.3 × 0.24 × 0.18 mm
*F*(000) = 846.676	

### Bis(acetylacetonato-κ2O,O')(2-amino-1-methyl-1H-\ benzimidazole-κN3)copper(II) Data collection

**Table:**

XtaLAB Synergy, Single source at home/near, HyPix3000 diffractometer	1693 reflections with *I*≥ 2σ(*I*)
Detector resolution: 10.0000 pixels mm^-1^	*R*_int_ = 0.066
ω scans	θ_max_ = 71.5°, θ_min_ = 4.2°
Absorption correction: multi-scan (CrysAlisPro; Rigaku OD, 2020)	*h* = −11→10
*T*_min_ = 0.517, *T*_max_ = 1.000	*k* = −17→17
19729 measured reflections	*l* = −19→19
2050 independent reflections	

### Bis(acetylacetonato-κ2O,O')(2-amino-1-methyl-1H-\ benzimidazole-κN3)copper(II) Refinement

**Table:**

Refinement on *F*^2^	Hydrogen site location: mixed
Least-squares matrix: full	H atoms treated by a mixture of independent and constrained refinement
*R*[*F*^2^ > 2σ(*F*^2^)] = 0.036	*w* = 1/[σ^2^(*F*_o_^2^) + (0.0585*P*)^2^ + 0.436*P*] where *P* = (*F*_o_^2^ + 2*F*_c_^2^)/3
*wR*(*F*^2^) = 0.109	(Δ/σ)_max_ = 0.001
*S* = 1.04	Δρ_max_ = 0.27 e Å^−^^3^
2050 reflections	Δρ_min_ = −0.33 e Å^−^^3^
146 parameters	Extinction correction: SHELXL2016/6 (Sheldrick 2015), Fc^*^=kFc[1+0.001xFc^2^λ^3^/sin(2θ)]^-1/4^
0 restraints	Extinction coefficient: 0.00034 (14)

### Bis(acetylacetonato-κ2O,O')(2-amino-1-methyl-1H-\ benzimidazole-κN3)copper(II) Special details

**Table:**

Geometry. All esds (except the esd in the dihedral angle between two l.s. planes) are estimated using the full covariance matrix. The cell esds are taken into account individually in the estimation of esds in distances, angles and torsion angles; correlations between esds in cell parameters are only used when they are defined by crystal symmetry. An approximate (isotropic) treatment of cell esds is used for estimating esds involving l.s. planes.

### Bis(acetylacetonato-κ2O,O')(2-amino-1-methyl-1H-\ benzimidazole-κN3)copper(II) Fractional atomic coordinates and isotropic or equivalent isotropic displacement parameters (Å^2^)

**Table:**

	*x*	*y*	*z*	*U*_iso_*/*U*_eq_	
Cu1	0.58425 (4)	0.250000	0.42118 (3)	0.04582 (19)	
O1	0.66063 (18)	0.34444 (12)	0.49868 (10)	0.0623 (4)	
N1	0.3473 (2)	0.250000	0.45198 (15)	0.0466 (6)	
O2	0.56911 (18)	0.34360 (12)	0.33081 (10)	0.0615 (4)	
N2	0.2624 (3)	0.250000	0.31270 (17)	0.0819 (10)	
H2A	0.350932	0.250000	0.292910	0.098*	
H2B	0.187784	0.250000	0.279310	0.098*	
C3	0.2406 (3)	0.250000	0.39601 (18)	0.0492 (7)	
C7A	0.2752 (3)	0.250000	0.52873 (17)	0.0440 (6)	
C8	−0.0378 (5)	0.250000	0.3850 (3)	0.0828 (14)	
C3A	0.1225 (3)	0.250000	0.5159 (2)	0.0507 (7)	
C4B	0.6857 (3)	0.43114 (19)	0.48236 (19)	0.0713 (7)	
C5	0.0806 (4)	0.250000	0.6613 (3)	0.0798 (12)	
H5	0.017475	0.250000	0.707242	0.096*	
C2B	0.5995 (3)	0.4322 (2)	0.33641 (19)	0.0724 (7)	
N3	0.1028 (3)	0.250000	0.43016 (16)	0.0534 (7)	
C3B	0.6587 (4)	0.4754 (2)	0.4067 (2)	0.0907 (10)	
H3B	0.682585	0.539925	0.402560	0.109*	
C4	0.0195 (4)	0.250000	0.5807 (2)	0.0701 (10)	
H4	−0.082124	0.250000	0.571234	0.105*	
C5B	0.7560 (5)	0.4878 (3)	0.5527 (2)	0.1149 (14)	
H5BA	0.760146	0.554218	0.537488	0.172*	
H5BB	0.854430	0.464517	0.562579	0.172*	
H5BC	0.697939	0.480611	0.602556	0.172*	
C6	0.2322 (4)	0.250000	0.6747 (2)	0.0671 (9)	
H6	0.267621	0.250000	0.729239	0.080*	
C7	0.3321 (4)	0.250000	0.6096 (2)	0.0550 (7)	
H7	0.433621	0.250000	0.619289	0.066*	
C1B	0.5684 (6)	0.4893 (2)	0.2591 (2)	0.1206 (15)	
H1BA	0.574813	0.556314	0.271846	0.181*	
H1BB	0.470795	0.474659	0.239196	0.181*	
H1BC	0.639781	0.473498	0.216864	0.181*	
H8A	−0.049 (6)	0.199 (3)	0.353 (3)	0.19 (2)*	
H8B	−0.100 (8)	0.250000	0.420 (4)	0.14 (3)*	

### Bis(acetylacetonato-κ2O,O')(2-amino-1-methyl-1H-\ benzimidazole-κN3)copper(II) Atomic displacement parameters (Å^2^)

**Table:**

	*U*^11^	*U*^22^	*U*^33^	*U*^12^	*U*^13^	*U*^23^
Cu1	0.0410 (3)	0.0525 (3)	0.0439 (3)	0.000	0.00055 (16)	0.000
O1	0.0659 (10)	0.0630 (10)	0.0580 (9)	0.0026 (8)	−0.0107 (7)	−0.0074 (8)
N1	0.0368 (12)	0.0605 (14)	0.0426 (12)	0.000	0.0026 (10)	0.000
O2	0.0728 (11)	0.0612 (10)	0.0505 (9)	−0.0109 (7)	0.0010 (7)	0.0085 (7)
N2	0.0433 (16)	0.160 (3)	0.0422 (14)	0.000	−0.0035 (12)	0.000
C3	0.0360 (14)	0.0645 (18)	0.0471 (15)	0.000	0.0009 (12)	0.000
C7A	0.0464 (15)	0.0420 (13)	0.0435 (14)	0.000	0.0033 (12)	0.000
C8	0.041 (2)	0.137 (5)	0.070 (3)	0.000	−0.0072 (19)	0.000
C3A	0.0441 (15)	0.0556 (17)	0.0524 (17)	0.000	0.0055 (13)	0.000
C4B	0.0746 (17)	0.0566 (14)	0.0828 (18)	0.0047 (12)	−0.0081 (14)	−0.0184 (13)
C5	0.071 (3)	0.114 (3)	0.055 (2)	0.000	0.0223 (17)	0.000
C2B	0.0847 (19)	0.0593 (15)	0.0731 (17)	−0.0010 (13)	0.0100 (14)	0.0128 (13)
N3	0.0374 (13)	0.0733 (18)	0.0494 (14)	0.000	−0.0018 (10)	0.000
C3B	0.129 (3)	0.0480 (14)	0.095 (2)	−0.0085 (16)	−0.009 (2)	−0.0014 (14)
C4	0.050 (2)	0.097 (3)	0.064 (2)	0.000	0.0117 (15)	0.000
C5B	0.148 (4)	0.078 (2)	0.119 (3)	0.000 (2)	−0.038 (3)	−0.039 (2)
C6	0.073 (2)	0.083 (2)	0.0449 (16)	0.000	0.0051 (15)	0.000
C7	0.0554 (19)	0.0618 (19)	0.0477 (16)	0.000	−0.0022 (14)	0.000
C1B	0.187 (4)	0.083 (2)	0.092 (3)	−0.013 (2)	−0.004 (2)	0.036 (2)

### Bis(acetylacetonato-κ2O,O')(2-amino-1-methyl-1H-\ benzimidazole-κN3)copper(II) Geometric parameters (Å, º)

**Table:**

Cu1—O1^i^	1.9378 (16)	C3A—C4	1.393 (5)
Cu1—O1	1.9378 (16)	C4B—C3B	1.381 (4)
Cu1—N1	2.196 (2)	C4B—C5B	1.516 (4)
Cu1—O2	1.9546 (16)	C5—H5	0.9300
Cu1—O2^i^	1.9546 (16)	C5—C4	1.404 (6)
O1—C4B	1.260 (3)	C5—C6	1.385 (5)
N1—C3	1.316 (4)	C2B—C3B	1.383 (4)
N1—C7A	1.390 (3)	C2B—C1B	1.498 (4)
O2—C2B	1.272 (3)	C3B—H3B	0.9300
N2—H2A	0.8600	C4—H4	0.9300
N2—H2B	0.8600	C5B—H5BA	0.9600
N2—C3	1.348 (4)	C5B—H5BB	0.9600
C3—N3	1.360 (4)	C5B—H5BC	0.9600
C7A—C3A	1.395 (4)	C6—H6	0.9300
C7A—C7	1.392 (4)	C6—C7	1.378 (5)
C8—N3	1.461 (5)	C7—H7	0.9300
C8—H8A	0.89 (5)	C1B—H1BA	0.9600
C8—H8A^i^	0.89 (5)	C1B—H1BB	0.9600
C8—H8B	0.79 (7)	C1B—H1BC	0.9600
C3A—N3	1.383 (4)		
			
O1^i^—Cu1—O1	85.85 (10)	C3B—C4B—C5B	119.4 (3)
O1—Cu1—N1	101.73 (7)	C4—C5—H5	119.0
O1^i^—Cu1—N1	101.73 (7)	C6—C5—H5	119.0
O1—Cu1—O2	92.44 (7)	C6—C5—C4	122.0 (3)
O1^i^—Cu1—O2^i^	92.44 (7)	O2—C2B—C3B	124.4 (3)
O1—Cu1—O2^i^	162.56 (7)	O2—C2B—C1B	114.8 (3)
O1^i^—Cu1—O2	162.56 (7)	C3B—C2B—C1B	120.8 (3)
O2—Cu1—N1	95.61 (7)	C3—N3—C8	126.6 (3)
O2^i^—Cu1—N1	95.61 (7)	C3—N3—C3A	106.3 (2)
O2^i^—Cu1—O2	84.01 (10)	C3A—N3—C8	127.1 (3)
C4B—O1—Cu1	125.89 (18)	C4B—C3B—C2B	125.9 (3)
C3—N1—Cu1	124.14 (19)	C4B—C3B—H3B	117.1
C3—N1—C7A	105.0 (2)	C2B—C3B—H3B	117.1
C7A—N1—Cu1	130.90 (19)	C3A—C4—C5	114.9 (3)
C2B—O2—Cu1	125.76 (18)	C3A—C4—H4	122.5
H2A—N2—H2B	120.0	C5—C4—H4	122.5
C3—N2—H2A	120.0	C4B—C5B—H5BA	109.5
C3—N2—H2B	120.0	C4B—C5B—H5BB	109.5
N1—C3—N2	124.5 (3)	C4B—C5B—H5BC	109.5
N1—C3—N3	113.4 (3)	H5BA—C5B—H5BB	109.5
N2—C3—N3	122.1 (3)	H5BA—C5B—H5BC	109.5
N1—C7A—C3A	109.5 (3)	H5BB—C5B—H5BC	109.5
N1—C7A—C7	130.4 (3)	C5—C6—H6	119.0
C7—C7A—C3A	120.1 (3)	C7—C6—C5	122.1 (3)
N3—C8—H8A^i^	112 (3)	C7—C6—H6	119.0
N3—C8—H8A	112 (3)	C7A—C7—H7	121.3
N3—C8—H8B	105 (5)	C6—C7—C7A	117.4 (3)
H8A—C8—H8A^i^	108 (6)	C6—C7—H7	121.3
H8A—C8—H8B	109 (4)	C2B—C1B—H1BA	109.5
H8B—C8—H8A^i^	109 (4)	C2B—C1B—H1BB	109.5
N3—C3A—C7A	105.9 (2)	C2B—C1B—H1BC	109.5
N3—C3A—C4	130.7 (3)	H1BA—C1B—H1BB	109.5
C4—C3A—C7A	123.5 (3)	H1BA—C1B—H1BC	109.5
O1—C4B—C3B	125.5 (3)	H1BB—C1B—H1BC	109.5
O1—C4B—C5B	115.1 (3)		
			
Cu1—O1—C4B—C3B	2.1 (4)	C3—N1—C7A—C7	180.000 (1)
Cu1—O1—C4B—C5B	−175.7 (2)	C7A—N1—C3—N2	180.000 (1)
Cu1—N1—C3—N2	0.000 (1)	C7A—N1—C3—N3	0.000 (1)
Cu1—N1—C3—N3	180.000 (1)	C7A—C3A—N3—C3	0.000 (1)
Cu1—N1—C7A—C3A	180.000 (1)	C7A—C3A—N3—C8	180.000 (1)
Cu1—N1—C7A—C7	0.000 (1)	C7A—C3A—C4—C5	0.000 (1)
Cu1—O2—C2B—C3B	4.8 (4)	C3A—C7A—C7—C6	0.000 (1)
Cu1—O2—C2B—C1B	−176.0 (2)	C5—C6—C7—C7A	0.000 (1)
O1—C4B—C3B—C2B	0.3 (6)	N3—C3A—C4—C5	180.000 (1)
N1—C3—N3—C8	180.000 (1)	C4—C3A—N3—C3	180.000 (1)
N1—C3—N3—C3A	0.000 (1)	C4—C3A—N3—C8	0.000 (1)
N1—C7A—C3A—N3	0.000 (1)	C4—C5—C6—C7	0.000 (1)
N1—C7A—C3A—C4	180.000 (1)	C5B—C4B—C3B—C2B	178.0 (4)
N1—C7A—C7—C6	180.000 (1)	C6—C5—C4—C3A	0.000 (1)
O2—C2B—C3B—C4B	−4.1 (6)	C7—C7A—C3A—N3	180.000 (1)
N2—C3—N3—C8	0.000 (1)	C7—C7A—C3A—C4	0.000 (1)
N2—C3—N3—C3A	180.000 (1)	C1B—C2B—C3B—C4B	176.9 (4)
C3—N1—C7A—C3A	0.000 (1)		

Symmetry code: (i) *x*, −*y*+1/2, *z*.

### Bis(acetylacetonato-κ2O,O')(2-amino-1-methyl-1H-\ benzimidazole-κN3)copper(II) Hydrogen-bond geometry (Å, º)

*Cg*1 is the centroid of the N1/C2/N3/C3*A*/C7*A* ring.

**Table:**

*D*—H···*A*	*D*—H	H···*A*	*D*···*A*	*D*—H···*A*
N2—H2*A*···O2	0.86	2.44	3.077 (3)	131
N2—H2*B*···O2^ii^	0.86	2.44	3.167 (3)	142
C5*B*—H5*BA*···*Cg*1^iii^	0.96	2.74	3.682 (4)	166

Symmetry codes: (ii) *x*−1/2, −*y*+1/2, −*z*+1/2; (iii) −*x*+1, *y*+1/2, −*z*+1.
